# Multifeature endoscopic swallowing patterns associated with pneumonia in hospitalized geriatric patients: a four-year longitudinal cohort study

**DOI:** 10.1186/s12877-026-07803-1

**Published:** 2026-06-16

**Authors:** Thomas D. Kocar, Sara Peranovic, Bendix Labeit, Sriramya Lapa, Paul Muhle, Sonja Suntrup-Krueger, Tineke Greiner, Julian Minor, Rainer Dziewas, Kiril Stoev, Nina Rosa Neuendorff, Rainer Wirth, Maryam Pourhassan, Gero Lueg

**Affiliations:** 1 Institute for Geriatric Research Ulm, Ulm University Medical Center, Ulm, Germany; 2 Department of Geriatric Medicine, Marien Hospital Herne, Ruhr University Bochum, Hölkeskampring 40, Herne, D-44625 Germany; 3 Department of Neurology, University Hospital Düsseldorf, Düsseldorf, Germany; 4 Department of Neurology, University Hospital Frankfurt, Goethe University Frankfurt, Frankfurt am Main, Germany; 5 Department of Neurology, University of Münster, Albert-Schweitzer-Campus 1, Building A1, 48149 Münster, Germany; 6 Department of Neurology, BG University Hospital Bergmannsheil, Ruhr University Bochum, Bochum, Germany; 7 Department of Neurology and Neurorehabilitation, Klinikum Osnabrück, Osnabrück, Germany; 8 Faculty of Medicine, Ruhr University Bochum, Bochum, Germany

**Keywords:** Oropharyngeal dysphagia, Geriatric patients, Pneumonia, Risk score, FEES, Fiberoptic endoscopic evaluation of swallowing

## Abstract

**Background:**

Oropharyngeal dysphagia is common in geriatric patients and a major risk factor for pneumonia. Fiberoptic endoscopic evaluation of swallowing (FEES) can identify specific swallowing abnormalities; however, apart from aspiration, the relationship of these abnormalities to pneumonia risk is not well understood. This study aimed to identify FEES-based swallowing abnormalities associated with long-term pneumonia risk beyond airway invasion alone and to develop and internally evaluate a transparent multifeature risk score.

**Methods:**

In this retrospective cohort study, 98 geriatric patients underwent FEES. Nine predefined FEES-derived swallowing features were analyzed using a clustering approach to identify a multifeature constellation associated with pneumonia. In addition, three methods were tested to develop a scoring system: clustering-based feature selection, penalized logistic regression, and a weighted ensemble of decision tree stumps, all assessed using fivefold cross-validation.

**Results:**

The clustering approach revealed a swallowing pattern comprising prolonged oral phase (excluding bread), delayed swallow reflex, reduced whiteout intensity, repetitive swallowing (excluding bread), piriform sinus residue, and airway invasion at PAS ≥ 3. A simple scoring system assigning one point per feature yielded an area under the receiver operating characteristic curve of 0.73 (95% CI 0.61–0.82). Each additional deficit increased pneumonia risk (odds ratio 1.82, 95% CI 1.24–2.67). The optimal Youden-optimized cut-off was ≥ 4 deficits, yielding a sensitivity of 0.46 (95% CI 0.29–0.63) and specificity of 0.89 (95% CI 0.79–0.96).

**Conclusions and Implications:**

Pneumonia risk in hospitalized geriatric patients with oropharyngeal dysphagia arises from the accumulation of functional swallowing impairments rather than isolated endoscopic findings. A transparent, exclusively FEES-based multifeature score provides a pragmatic framework for risk stratification.

**Supplementary Information:**

The online version contains supplementary material available at 10.1186/s12877-026-07803-1.

## Introduction

Oropharyngeal dysphagia (OD) is a common geriatric syndrome and a major health concern in older adults, characterized by multifactorial and heterogeneous swallowing impairments, and associated with adverse outcomes including malnutrition, pneumonia, and increased mortality in both hospitalized and community-dwelling populations [1–3]. In geriatric patients with dysphagia, pneumonia is frequently diagnosed without a clinically documented aspiration event, and aspiration pneumonia is not routinely labeled despite a high prevalence of swallowing impairment [4, 5]. Prospective geriatric cohort data demonstrate that dysphagia predicts pneumonia irrespective of etiologic classification, indicating that a strict distinction between aspiration pneumonia and other pneumonia entities is often not feasible in real-world clinical practice [4].

VFSS studies in older adults have reported heterogeneous findings, including silent aspiration or no observable airway compromise during standardized assessment, which complicates causal attribution of pneumonia to aspiration alone [6]. Meta-analytic evidence confirms an increased pneumonia risk associated with dysphagia across care settings, despite heterogeneity in pneumonia definitions and diagnostic approaches [2, 7]. Previous studies have used different swallowing-related approaches to estimate pneumonia risk. Clinical and multiconsistency swallowing assessments have identified dysphagia as a general risk factor for community-acquired pneumonia and pneumonia readmission in older adults [4, 6, 8]. Instrumental studies have frequently focused on aspiration or unsafe swallowing as prognostically relevant findings, whereas VFSS-based analyses have examined selected physiological parameters such as laryngeal vestibule closure, hyoid movement, oral transit time, or pharyngeal residue [7, 9–11].

Other models and reviews have emphasized that pneumonia risk is also shaped by non-swallowing markers of systemic vulnerability, including functional dependence, comorbidity, cognitive impairment, malnutrition, and frailty [4, 7, 8]. Poor oral health and colonization of the oral microbiome by potentially pathogenic microorganisms have also been associated with aspiration pneumonia risk in older people [12]. However, these approaches either rely on clinical screening, selected instrumental parameters, or combined clinical-instrumental models. These specific contribution of cumulative FEES-derived swallowing abnormalities across several functionally relevant components of swallowing therefore remains insufficiently defined in hospitalized geriatric patients.

Swallowing is a complex sensorimotor process involving multiple interacting components, and pneumonia in multimorbid geriatric patients rarely results from a single abnormality alone. Therefore, the aim of the present study was to identify FEES-based swallowing abnormalities associated with subsequent pneumonia in hospitalized geriatric patients with OD, with particular focus on features beyond airway invasion alone. Based on these findings, we sought to develop and internally evaluate a transparent, exclusively FEES-based multifeature score for pneumonia risk stratification.

## Methods

### Study design and population

This retrospective longitudinal cohort study was conducted at an acute geriatric care unit of Marien Hospital Herne, Germany, and represents an extended analysis of endoscopic swallowing parameters from a previously published cohort [13]. Consecutive patients who underwent flexible endoscopic evaluation of swallowing (FEES) during admission to the geriatric ward between December 2018 and January 2020 were included. Inclusion criteria comprised patients aged ≥ 65 years with documented FEES and a diagnosis of oropharyngeal dysphagia at endoscopic assessment. Exclusion criteria were incomplete baseline or follow-up data and refusal to participate in the written follow-up questionnaire or structured telephone interview; consequently, no missing data were present.

The primary outcome was the occurrence of pneumonia during follow-up. Secondary outcomes included recurrent pneumonia, time to first pneumonia, and all-cause mortality.

### Geriatric and FEES assessments

At hospital admission, patients underwent routine comprehensive geriatric assessment. Nutritional status was assessed with the Mini Nutritional Assessment–Short Form (MNA-SF) [14], functional independence with the Barthel Index [15], frailty with the FRAIL scale [16], and sarcopenia risk with the SARC-F questionnaire [17]. Handgrip strength was measured three times on the dominant or unaffected side, using the highest value [18]. Cognitive status was evaluated with the Montreal Cognitive Assessment (MoCA) [19]; if completion was limited by non-cognitive factors (e.g., visual impairment), scores were extrapolated from completed items, in line with established procedures [20].

FEES was performed by a physician and an experienced speech-language pathologist following a standardized protocol [21]. Recordings were scored in consensus by two trained raters using standardized criteria; for quality assurance, a random subset of videos (*n* = 15) was independently re-rated by two certified FEES experts blinded to clinical data and outcomes (interrater agreement was 85%). The testing sequence comprised standardized pudding-like consistency, nectar-thick and thin liquids (5–10 mL), and bite-sized bread, according to the International Dysphagia Diet Standardisation Initiative (IDDSI) [22]. If oral transport of bread was inadequate, water was added to form a mixed bolus. A placebo tablet and capsule were also swallowed with water to assess solid and mixed swallowing. Each consistency was tested separately, and the most severe finding per parameter was analyzed.

Dysphagia severity was rated using an established ordinal scale for neurogenic dysphagia in conjunction with the Penetration-Aspiration Scale (PAS) [23, 24]. Habitual oral intake prior to admission was assessed using the Functional Oral Intake Scale (FOIS) [25]. Swallowing function was analyzed in analogy to previous neurogenic dysphagia studies [13, 21], with study-specific extensions for oral phase parameters and repetitive swallowing to better capture complex geriatric swallowing patterns. At the beginning of FEES, a resting observation without bolus administration assessed secretion management and spontaneous swallowing; a spontaneous swallow was defined as ≥ 1 swallow within ~ 30 s [26]. Because the oral phase cannot be directly visualized by FEES, oral transit time was assessed indirectly as the interval between visible bolus entry into the oral cavity and initiation of the pharyngeal swallow and considered prolonged if > 3 s (liquids), > 5 s (semi-solids), or > 20 s (solids), based on VFSS-derived reference values [27]. Prolongation was coded as present if abnormal transit occurred in any tested consistency; pure solid bread was excluded from dichotomous analyses and analyzed descriptively or as part of mixed consistencies. Premature spillage was defined as pre-deglutitive bolus entry into the valleculae or below prior to swallow initiation. Delayed swallow reflex was defined as a latency > 3 s between bolus arrival in the valleculae and reflex initiation [28]. Swallow reflex triggering at the level of the piriform sinus was coded as caudally triggered. White-out intensity was rated by the proportion of endoscopic white coverage and classified as complete (> 2/3), moderately reduced (1/3–2/3), or severely reduced (< 1/3) [21]. Vallecular and piriform sinus residues were rated using the Yale Pharyngeal Residue Severity Rating Scale; scores ≥ 3 (residues occupying > 5% of the pharyngeal space) were classified as pathological [29]. Repetitive swallowing was defined as ≥ 2 consecutive clearing swallows within 5 s after the initial deglutition. For oral bolus transfer and post-swallow behavior (prolonged oral phase and repetitive swallowing), definitions were restricted to non-bread consistencies.

### Primary and secondary outcomes

Follow-up data were obtained using a structured multi-source outcome assessment based on the follow-up procedure used in our previously published cohort study [13]. Assessments were performed as a single cross-sectional survey within a defined study period, resulting in variable intervals between FEES and follow-up. The interview template has not been separately published or validated as a standalone instrument; therefore, an English-language version is provided as Supplementary Table S1. For all patients, internal hospital records were reviewed to identify documented pneumonia events, survival status, and date of death. In addition, structured follow-up interviews were conducted with patients or, when appropriate, relatives or proxies. The interview assessed whether pneumonia had occurred after hospital discharge, the approximate timing and number of pneumonia episodes, and whether the diagnosis had been made by a treating physician. Whenever available, general practitioners were contacted by telephone for confirmation or clarification. Pneumonia was classified as present if physician-diagnosed pneumonia was documented in internal hospital records, confirmed by the general practitioner, or reported by the patient or proxy during structured follow-up. This structured multi-source procedure resulted in complete outcome data for all included patients.

### Data analysis

All data analyses were performed using IBM SPSS Statistics, Version 31.0 (IBM Corp., Armonk, NY) and the Python programming language (Python Software Foundation). Descriptive statistics were used to summarize cohort characteristics, with continuous variables reported as median and interquartile range (IQR) and categorical variables as counts and percentages. Exploratory univariable logistic regression analyses were performed to assess the association between individual FEES-based parameters and pneumonia occurrence. Odds ratios (OR) with 95% confidence intervals are reported.

Our main analysis addressed two complementary objectives: First, we aimed to identify a multifeature endoscopic swallowing pattern associated with the subsequent development of pneumonia. To this end, hierarchical agglomerative clustering was applied within a predefined set of dichotomized FEES-derived parameters **(**Table 1). This feature pool was defined a priori by two certified FEES instructors within the author group before model development to cover several clinically relevant components of the swallowing process, including resting observation, oral bolus transfer, swallow triggering, pharyngeal clearance, and airway protection. Selection was based on physiological plausibility, prior FEES literature, clinical interpretability, and consistent availability within the standardized FEES protocol. Parameters not meeting these criteria, including isolated consistency-specific observations, were not included in the predefined feature pool.

**Table 1:** Predefined FEES-based endoscopic swallowing parameters and operational definitions used for data-driven analyses. Endoscopic swallowing parameters were defined a priori by two certified FEES instructors within the author group based on physiological plausibility, prior FEES literature, clinical interpretability, and consistent availability within the standardized FEES protocol. For parameters reflecting oral bolus transfer and post-swallow behavior (prolonged oral phase and repetitive swallowing), definitions were restricted to non-bread consistencies according to the predefined operational definitions. Abbreviations: FEES, flexible endoscopic evaluation of swallowing; PAS, Penetration–Aspiration Scale; YPRSRS, Yale Pharyngeal Residue Severity Rating Scale

FEES domain	Parameter	Definition
Resting observation	Spontaneous swallow	Occurrence within 30 s resting observation
Oral phase	Prolonged oral phase	Delayed bolus transfer in ≥ 1 non-solid or mixed consistency (pure bread excluded)
Pre-deglutitive phase	Premature bolus spillage	Presence of spillage before swallow initiation
	Delayed swallow reflex trigger	Latency > 3 s
	Swallow reflex trigger zone	Piriform sinus
Intra-deglutitive phase	Reduced whiteout intensity	< 1/3 pharyngeal whiteout coverage
Post-deglutitive phase	Repetitive swallowing	≥ 2 swallows after initial bolus swallow (excluding bread)
	Piriform sinus residue	YPRSRS ≥ 3
Airway invasion	Penetration / Aspiration	PAS ≥ 3

Patient data were represented as binary feature vectors, and Euclidean distance was used as the similarity metric. To identify the most informative feature subset, all possible feature combinations were evaluated, and the adjusted Rand index (ARI) was used as the optimization criterion, reflecting agreement between the derived cluster structure and pneumonia status. For clustering-based feature selection, hierarchical agglomerative clustering was used as an established exploratory approach to identify informative feature structures within the predefined FEES-derived feature pool [29, 30].

Second, we aimed to derive a clinically applicable FEES-based scoring system for pneumonia risk stratification. Three complementary modeling approaches were evaluated, representing increasing levels of model complexity [31]: (i) the clustering-based approach described above, (ii) penalized logistic regression using the least absolute shrinkage and selection operator (LASSO), and (iii) a supervised ensemble learning approach using adaptive boosting (AdaBoost) on decision tree stumps. The clustering-based approach relies on a distance-based representation with fixed feature contributions. LASSO regression introduces optimized feature weighting while controlling for multicollinearity, whereas AdaBoost additionally allows for automated threshold selection. In contrast to clustering and LASSO, which relied on expert-defined dichotomized inputs, the AdaBoost model operated on minimally preprocessed data and internally determined optimal split points. To ensure unbiased performance assessment, stratified fivefold cross-validation was applied across the full dataset. Within each fold, model performance was evaluated separately in the training and test sets and the mean across folds is reported. For the clustering approach, the feature subset achieving the highest ARI was combined into an additive score, with each pathological feature contributing one point. In the LASSO model, the regularization parameter was adjusted to retain a fixed number of five features, and model coefficients were transformed into integer weights using an iterative scaling procedure that preserved their relative contributions and optimized discrimination. The AdaBoost model was configured with a fixed number of five estimators, and its continuous weights were converted to integer values using the same scaling approach as in the LASSO model. Performance was primarily assessed using receiver operating characteristics (ROC) analyses, with the area under the curve (AUC) calculated for training and test sets. The best-performing models were subsequently refitted on the complete dataset. Final ROC curves were generated, and sensitivity and specificity were calculated across score thresholds. Optimal cut-off values were determined using the Youden index. Confidence intervals (CI) were obtained using bootstrap resampling with 1,000 iterations. All modeling analyses are reported in accordance with the TRIPOD Framework (Supplementary Table S2) [32].

Associations between FEES-based scores and clinical outcomes were evaluated using regression models adjusted for the predefined clinical covariates age, dementia status, Barthel Index at admission, handgrip strength, and nutritional status assessed by the MNA-SF. For binary outcomes (pneumonia occurrence and recurrent pneumonia), logistic regression models were fitted, and odds ratios (OR) with 95% CI were reported. For time-to-event outcomes (time to pneumonia and time to all-cause death), Cox proportional hazards regression was applied, and hazard ratios (HR) with 95% CI were calculated. For all analyses, a two-sided p value < 0.05 was considered statistically significant.

## Results

### Cohort characteristics

The study cohort comprised 98 geriatric inpatients in whom a clinical suspicion of OD was confirmed by FEES as part of the diagnostic work-up. Baseline demographic, functional, and clinical characteristics are summarized in (Table 2). Functional impairment and nutritional vulnerability were common. Median follow-up from FEES was 1,322 days [IQR 1,241–1,433]. During follow-up, 37 patients (37.8%) developed pneumonia (median time to event 1,273 days [IQR 1,123–1,397]), while 35 patients (35.7%) died before follow-up assessment. FEES-documented abnormalities across all swallowing phases are summarized in (Table 3). During resting observation, a spontaneous swallow within 30 s was present in approximately half of patients. Prolonged oral phase and premature bolus spillage were frequent findings, while delayed swallow reflex occurred in about one fifth of patients. In univariable analyses, prolonged oral phase, delayed swallow reflex, repetitive swallowing, piriform sinus residue, and aspiration at PAS ≥ 6 were associated with pneumonia, whereas PAS ≥ 3 showed no significant association.

**Table 2:** Study cohort characteristics. Continuous variables are reported as median with interquartile range (IQR) and categorical variables as number and percentage, where appropriate. Follow-up time intervals are reported relative to flexible endoscopic evaluation of swallowing (FEES). Abbreviations: FEES, flexible endoscopic evaluation of swallowing; MNA-SF, Mini Nutritional Assessment–Short Form; SARC-F, Strength, Assistance with walking, Rising from a chair, Climbing stairs, and Falls

Characteristic		Total cohort (*n* = 98)
Age, years, median [IQR]		81.5 [75.0–86.0]
Male sex, n (%)		66 (67.3)
Underlying disease	Cerebrovascular disease	27 (27.6)
	Parkinsonian syndrome	17 (17.3)
	Alzheimer’s disease	14 (14.3)
	Non-neurological medical disease	24 (24.5)
	No dysphagia-relevant underlying disease	16 (16.3)
Primary indication for FEES referral	Malnutrition	26 (26.5)
	Clinical assessment	24 (24.5)
	Known dysphagia	18 (18.4)
	Globus sensation / subjective dysphagia	18 (18.4)
	Caregiver-reported dysphagia	12 (12.2)
Geriatric assessment	MNA-SF score, median [IQR]	8 [6–10]
	Normal nutritional status, n (%)	7 (8)
	At risk of malnutrition, n (%)	44 (49)
	Malnourished, n (%)	39 (43)
	Barthel Index, median [IQR]	45 [35–60]
	Frail Simple score, median [IQR]	4 [3–4]
	SARC-F score, median [IQR]	7 [5–8]
	Hand grip strength, kg, median [IQR]	18.5 [11.8–28.0] (*n* = 86)
	Peak flow, L/min, median [IQR]	220 [150–300]
Follow-up data	Time from FEES to pneumonia, days, median [IQR]	1273.0 [1122.5–1397.0]
	Time from FEES to follow-up assessment, days, median [IQR]	1321.5 [1240.5–1433.0]
	Time from FEES to death, days, median [IQR]	895.5 [352.5–1290.5]
Follow-up outcomes	Pneumonia during follow-up, n (%)	37 (38)
	No pneumonia, n (%)	61 (62)
Number of pneumonias	0	61 (62)
	1	11 (11)
	≥ 2	26 (27)
Mortality, n (%)	Yes	35 (36)
	No	63 (64)

**Table 3:** Endoscopic swallowing characteristics and their association with pneumonia risk. Data are presented as n (%) and odds ratio (OR) with associated p-values from a univariable logistic regression. Spontaneous swallow was defined as ≥ 1 swallow during a ~ 30 s resting observation without bolus. Prolonged oral phase indicates pathological oral transit time in ≥ 1 tested consistency (pudding, liquids, or mixed bolus; bread excluded). Repetitive swallowing was defined as ≥ 2 clearing swallows within 5 s. Delayed swallow reflex was defined as latency > 3 s; caudal triggering as reflex initiation at the piriform sinus. Pathological residues were defined as YPRSRS ≥ 3, airway invasion as PAS ≥ 3, aspiration as PAS ≥ 6, and dietary restriction as FOIS < 7 before or after FEES.

Phase	Parameter	Total cohort (*n* = 98), n (%)	OR (Exp(B))	*p*-value
Resting state	No spontaneous swallow	51 (52.0%)	1.36 (0.60–3.08)	0.47
Oral phase	Prolonged oral phase	38 (38.8%)	2.81 (1.20–6.57)	**0.02**
	Repetitive swallowing	52 (53.1%)	2.62 (1.12–6.16)	**0.03**
Trigger phase	Delayed swallow reflex (> 3 s)	22 (22.4%)	5.26 (1.89–14.66)	**< 0.01**
	Caudal triggering (piriform sinus)	47 (48.0%)	1.77 (0.77–4.03)	0.18
Pre-deglutitive phase	Premature spillage (any consistency)	55 (56.1%)	1.49 (0.65–3.42)	0.35
Intra-deglutitive phase	White-out intensity (< 1/3 coverage)	43 (43.9%)	1.94 (0.85–4.45)	0.12
Post-deglutitive phase	Vallecular residues (YPRSRS) ≥ 3)	87 (88.8%)	1.71 (0.42–6.90)	0.45
	Piriform sinus residues (YPRSRS) ≥ 3)	53 (54.1%)	2.98 (1.25–7.09)	**0.01**
Airway protection	Airway invasion (PAS ≥ 3)	22 (22.4%)	0.74 (0.31–1.75)	0.49
	Aspiration (PAS ≥ 6)	16 (16.3%)	3.40 (1.12–10.32)	**0.03**
Dietary restrictions	FOIS before FEES < 7	23 (23.5%)	3.52 (1.33–9.28)	**0.01**
	FOIS after FEES < 7	64 (65.3%)	2.69 (1.06–6.84)	**0.04**

Abbreviations: *FEES *fiberoptic endoscopic evaluation of swallowing, *YPRSRS* Yale Pharyngeal Residue Severity Rating Scale, *PAS* Penetration–Aspiration Scale, *FOIS* Functional Oral Intake Scale

Bold *p*-values indicate statistical significance (*p* < 0.05)

### FEES-derived swallowing patterns associated with pneumonia

**Fig. 1:**
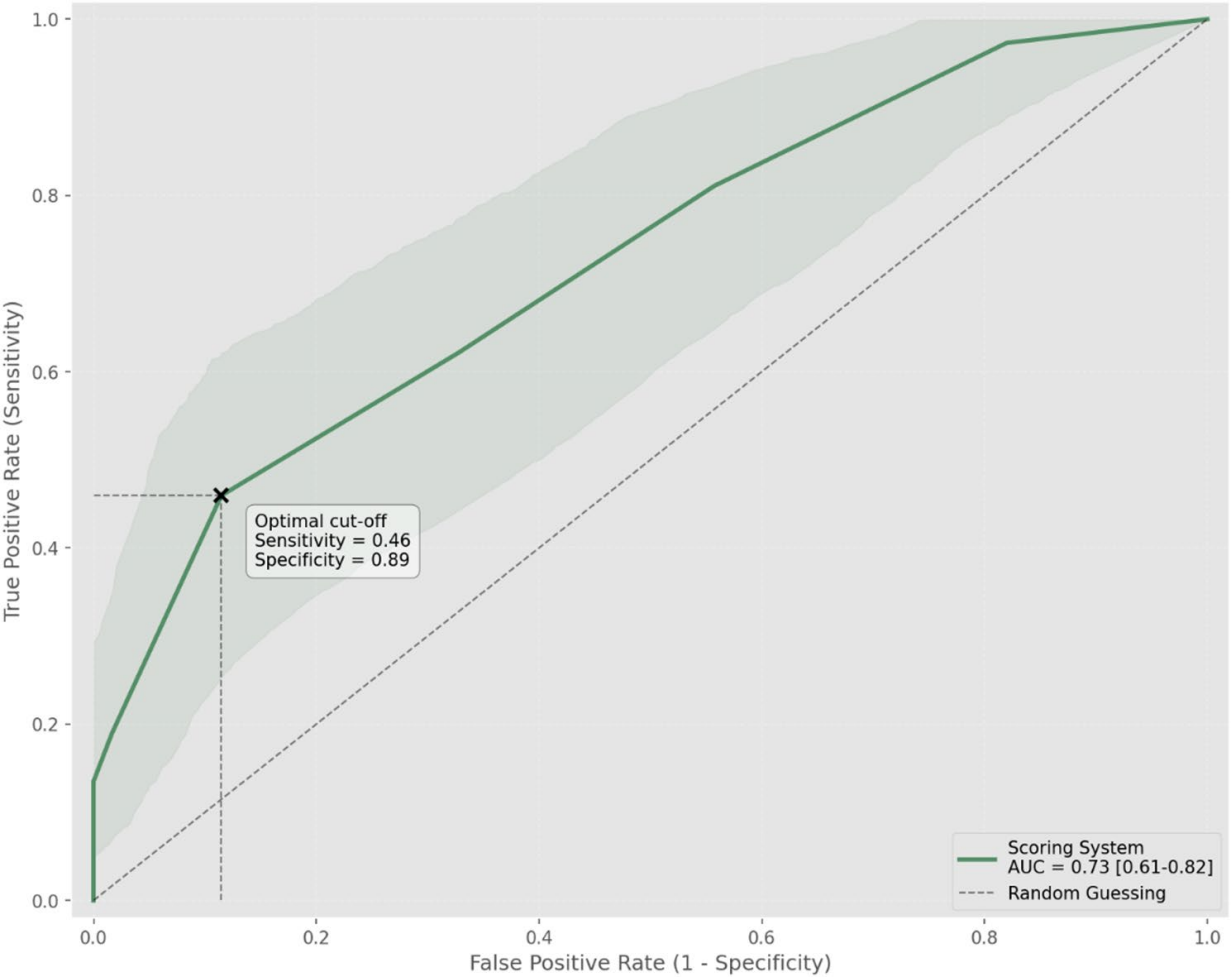
Receiver Operating Characteristics Curve for the Pneumonia Risk Scoring System. The area under the curve (AUC) is 0.73 (95% CI 0.61–0.82). The Youden-optimized cut-off is 4, with a sensitivity of 0.46 (95% CI 0.29–0.63) and specificity of 0.89 (95% CI 0.79–0.96). The shaded area indicates the 95% confidence interval of the curve, generated by bootstrapping with 1000 iterations and interpolation

Within the predefined, expert-selected FEES feature pool (Table 1), the clustering identified a discriminative subset comprising prolonged oral phase (excluding bread), delayed swallow reflex trigger (> 3s), reduced whiteout intensity, repetitive swallowing (excluding bread), piriform sinus residue (YPRSRS ≥ 3), and penetration–aspiration (PAS ≥ 3). Based on this set, an additive FEES-based pneumonia risk score ranging from 0 to 6 points was constructed (Table 4). ROC analysis demonstrated moderate discriminative performance for pneumonia (Fig. 1, AUC 0.73, 95% CI 0.61–0.82). The Youden-optimal threshold was ≥ 4 points, yielding a sensitivity of 0.46 (95% CI 0.29–0.61) and a specificity of 0.89 (95% CI 0.80–0.96). In covariate-adjusted regression models, each one-point increase in the clustering score was associated with higher odds of pneumonia (OR 1.82, 95% CI 1.24–2.67; *p* < 0.01) and recurrent pneumonia (OR 2.01, 95% CI 1.31–3.09; *p* < 0.01). In similarly adjusted time-to-event analyses, higher clustering scores were associated with an increased hazard of first pneumonia (HR 1.48, 95% CI 1.16–1.89; *p* < 0.01), whereas the association with mortality did not reach statistical significance (HR 1.21, 95% CI 0.99–1.46; *p* = 0.06; Supplementary Table S3).

**Table 4:** Scoring system for pneumonia risk prediction. Each of the six FEES-derived features contributes exactly 1 point, yielding a total score ranging from 0 to 6 points. Increasing scores were associated with a higher risk of pneumonia (odds ratio per point increase: 1.82; 95% CI 1.24–2.67; *p* < 0.01). A threshold of ≥ 4 points demonstrated the best overall performance based on the Youden Index, with a sensitivity of 0.46 (95% CI 0.29–0.61) and a specificity of 0.89 (95% CI 0.80–0.96). Abbreviations: FEES, flexible endoscopic evaluation of swallowing; PAS, Penetration–Aspiration Scale; YPRSRS, Yale Pharyngeal Residue Severity Rating Scale

FEES parameter	Points
Prolonged oral phase (excluding bread)	1
Delayed swallow reflex trigger (> 3 s)	1
Reduced white-out intensity (< 1/3)	1
Repetitive swallowing (excluding bread, ≥ 2)	1
Piriform sinus residue (YPRSRS ≥ 3)	1
Penetration–aspiration (PAS ≥ 3)	1

In the 5-fold cross-validation, the clustering approach described above showed comparable training and test performance (ROC AUC 0.69 vs. 0.66), indicating no significant overfitting. Complementary modeling approaches did not improve performance; LASSO selected largely overlapping features (Supplementary Tables S4 and S5) with ROC AUC 0.74 in training and 0.61 in test data, whereas AdaBoost showed signs of substantial overfitting (0.76 in training, 0.58 in test data). The clustering-derived pneumonia risk score thus constituted the definitive scoring system.

## Discussion

In this 4-year longitudinal cohort of hospitalized geriatric patients with FEES-confirmed OD, a multifeature endoscopic swallowing pattern was associated with the occurrence, recurrence, and time to pneumonia. A score operationalizing this pattern demonstrated a pronounced and continuous risk gradient, indicating that pneumonia risk increases progressively with the accumulation of swallowing-related abnormalities rather than emerging from a single endoscopic marker such as airway invasion alone.

Exploratory univariable analyses of individual FEES parameters (Table 3) showed that prolonged oral phase, delayed swallow reflex, repetitive swallowing, piriform sinus residue, and aspiration at a higher PAS cutoff (PAS ≥ 6) were associated with pneumonia, whereas a lower cutoff (PAS ≥ 3) was not. Normative FEES data indicate that low-grade penetration, and occasionally even aspiration, may also occur in healthy older adults [33]. This underscores the need to interpret lower-threshold airway invasion findings in clinical context and supports the rationale for considering additional swallowing abnormalities beyond PAS cut-offs alone. These findings are consistent with the thresholds used in the clustering-derived score and illustrate the stepwise contribution of multiple swallowing abnormalities to pneumonia risk.

Within a neurogeriatric framework, OD is conceptualized as a multi-etiological syndrome characterized by heterogeneous functional impairments rather than a uniform, single-marker condition [34]. Consistent with this concept, our findings indicate that pneumonia risk emerges from the accumulation of endoscopic abnormalities across different phases of the swallowing process rather than from any isolated instrumental finding. Consistent with previous cohort and meta-analytic evidence, OD has been shown to be independently associated with community-acquired pneumonia and pneumonia readmission in older adults, even after adjustment for comorbidity and functional status [2, 4, 6]. Although airway invasion remains clinically relevant and has been linked to pneumonia and mortality, available evidence also highlights the contribution of additional functional impairments, including delayed swallow initiation and impaired bolus clearance with residue burden, to pneumonia risk [9–11]. In this context, airway invasion should be understood as a downstream manifestation of underlying swallowing dysfunction rather than as a distinct swallowing phenotype, with FEES providing insight into cumulative functional vulnerability beyond the detection of single aspiration events [21, 28].

Accordingly, the present analysis focused on identifying endoscopic features across different swallowing phases that consistently co-occur in patients who subsequently develop pneumonia. This pneumonia-associated feature constellation was operationalized as a transparent, unweighted additive FEES score ranging from 0 to 6, reflecting abnormalities across distinct phases of the swallowing sequence rather than the ordinal severity of a single mechanism. The score components were dichotomized using predefined, clinically grounded thresholds and combined to capture the cumulative burden of functionally relevant impairments along oral bolus processing, swallow initiation, pharyngeal clearance, and airway protection (Table 4). In contrast to prior instrumental scoring systems that rely on cut-offs, ordinal severity grading, or clinical and demographic variables, the present score is derived exclusively from FEES parameters. Despite this, it demonstrates comparable predictive performance with an ROC AUC of 0.73, matching values reported in the literature [10, 35, 36]. Within this framework, the Youden-derived cut-off of ≥ 4 should be viewed as a pragmatic classification aid rather than a fixed threshold, supporting risk stratification rather than binary classification (Fig. 1, Supplementary Table S6). The cut-off showed high specificity but limited sensitivity, indicating that the score should not be used as a screening tool to exclude future pneumonia risk. Rather, it may help identify a subgroup of geriatric patients with a particularly high FEES-derived risk profile who may warrant closer clinical attention and preventive management.

The robustness of the identified swallowing pattern was evaluated using a sequential analytical strategy balancing interpretability and parsimony. From a predefined set of nine FEES-derived features, unsupervised clustering identified a pneumonia-associated constellation based solely on endoscopic findings. Feature relevance and reduction to a minimal parameter set were then examined using LASSO logistic regression, which confirmed the same core components without altering the primary pattern (Supplementary Table S4). As a sensitivity analysis, a more flexible supervised learning approach (AdaBoost) was evaluated but did not yield additional clinically meaningful insights.

Taken together, these findings support the interpretation that the observed associations reflect a stable, FEES-derived swallowing pattern. At the continuous level, increasing score values were associated with a higher likelihood and earlier occurrence of pneumonia, indicating a clear risk gradient across the score range (Supplementary Table S6). In contrast, the association with all-cause mortality was weaker, suggesting that the score primarily captures dysphagia-related pneumonia risk rather than overall disease burden. These findings differentiate the present approach from prior pneumonia risk scores that combine swallowing parameters with markers of multimorbidity or systemic illness, such as the model proposed by Kim and colleagues [10]. Instead, risk stratification in this cohort was defined exclusively by FEES-derived swallowing abnormalities, supporting a mechanism-oriented, dysphagia-specific risk construct.

### Limitations

Several limitations should be acknowledged. The retrospective single-center design limits causal inference and generalizability, and external validation is warranted. The score was intentionally constructed as a simple additive measure reflecting cumulative swallowing impairment; given the sample size and number of pneumonia events, more complex feature weighting was not pursued, and the score should be interpreted as a pragmatic risk stratification tool rather than a fully optimized prediction model. Pneumonia outcomes were based on structured follow-up and medical record review, precluding differentiation between community-acquired and aspiration pneumonia; diagnoses were not radiologically confirmed, and some misclassification cannot be excluded. Furthermore, the follow-up interval was relatively long and variable. Pneumonia events occurring late after the index FEES cannot be causally attributed to the baseline swallowing pattern alone, because swallowing function, frailty, intercurrent illness, oral health, and care conditions may change over time. Finally, the proposed threshold (≥ 4) of the pneumonia risk scoring system showed higher specificity than sensitivity, supporting its use for risk stratification rather than screening.

## Conclusion

In hospitalized geriatric patients with OD, pneumonia risk is best understood as the result of accumulating functional swallowing impairments rather than a single endoscopic abnormality. A transparent, exclusively FEES-based multifeature score provides a pragmatic framework for operationalizing this risk without reliance on disease-specific or multimorbidity markers.

## Supplementary information

Supplementary Material 1.

## Data Availability

The datasets analyzed during the current study are available from the corresponding author on reasonable request. All analysis scripts used for clustering and statistical evaluation are openly available (GitHub repository: https://github.com/IfGF-UUlm/dysphagia-pneumonia).

## Abbreviations

AdaBoost: Adaptive Boosting
ARI: Adjusted Rand Index
AUC: Area Under the Curve
BI: Barthel Index
CI: Confidence Interval
EWGSOP2: European Working Group on Sarcopenia in Older People 2
FEES: Flexible Endoscopic Evaluation of Swallowing
FOIS: Functional Oral Intake Scale
HR: Hazard Ratio
IDDSI: International Dysphagia Diet Standardisation Initiative
IQR: Interquartile Range
LASSO: Least Absolute Shrinkage and Selection Operator
MNA-SF: Mini Nutritional Assessment – Short Form
MoCA: Montreal Cognitive Assessment
OD: Oropharyngeal Dysphagia
OR: Odds Ratio
PAS: Penetration–Aspiration Scale
ROC: Receiver Operating Characteristic
SARC-F: Strength, Assistance with walking, Rising from a chair, Climbing stairs, and Falls questionnaire
SD: Standard Deviation
SLP: Speech–Language Pathologist
STROBE: STrengthening the Reporting of Observational studies in Epidemiology
TRIPOD: Transparent Reporting of a multivariable prediction model for Individual Prognosis or Diagnosis
VFSS: Videofluoroscopic Swallowing Study
YPRSRS: Yale Pharyngeal Residue Severity Rating Scale
